# Evaluated Wound Healing Property of *Aloe vera*-Coated Dextrane Sulfate/Chitosan Nanoparticles Encapsulating *Eucalyptus* in a Rat Model

**DOI:** 10.3390/pharmaceutics18070873

**Published:** 2026-07-17

**Authors:** Ebtesam A. Mohamad, Amany M. Gad, Rana H. Abd El-Rhman, Mona S. Elneklawi, Mirhane Mostafa Darwish

**Affiliations:** 1 Radiology and Medical Imaging Department, College of Applied Medical Sciences, Prince Sattam Bin Abdul-Aziz University, Al-Kharj 11942, Saudi Arabia; 2 Biophysics Department, Faculty of Science, Cairo University, Giza 12613, Egypt; mirhane@sci.cu.edu.eg; 3 Department of Pharmacology and Toxicology, Faculty of Pharmacy, Sinai University-Kantara Branch, El Ismailia 41521, Egypt; amany.gad@su.edu.eg (A.M.G.); rana.ahmed@su.edu.eg (R.H.A.E.-R.); 4 Department of Pharmacology, Egyptian Drug Authority (EDA), Formerly NODCAR, Giza 12311, Egypt; 5 Biomedical Equipment Department, Faculty of Applied Medical Sciences, October 6 University, 6th of October City 12557, Egypt; mona.saadeldin.ams@o6u.edu.eg

**Keywords:** chitosan, *Aloe vera*, wound healing, *Eucalyptus*, nanoparticles

## Abstract

**Background/Objectives**: A hydrogel with enhanced therapeutic properties was developed using chitosan, dextran sulfate, and *Aloe vera*-coated nanoparticles encapsulating eucalyptus extract. **Methods**: The physicochemical characteristics of the synthesized nanoparticles were evaluated using transmission electron microscopy (TEM), scanning electron microscopy (SEM), dynamic light scattering (DLS), Fourier-transform infrared spectroscopy (FTIR), and antibacterial activity assays. The in vitro release profile of *Eucalyptus staigeriana* extract was assessed at physiological pH 7.4, and the in vivo wound healing performance was subsequently investigated in a rat model. **Results**: The results indicated that the nanocarrier system provided a controlled and sustained release of eucalyptus extract. *Aloe vera*-coated dextran sulfate/chitosan nanoparticles encapsulating *E. staigeriana* inhibited the growth of *Staphylococcus aureus* by 40%. Moreover, animals treated with *Aloe vera* @ DX/CS/EE nanoparticles exhibited markedly improved wound contraction, with mean reductions of 27.45%, 19.18%, 18.12%, and 7.03% compared with the control, DX/CS nanoparticles, *Aloe vera* @ DX/CS nanoparticles, and DX/CS/EE nanoparticles groups, respectively. Histological analysis further confirmed that treatment with *Aloe vera* @ DX/CS/EE nanoparticles led to superior tissue organization and accelerated dermal regeneration. **Conclusions**: Overall, the dextran sulfate/chitosan hydrogel coated with *Aloe vera* and loaded with eucalyptus extract demonstrates strong potential as an effective wound dressing material capable of enhancing healing outcomes.

## 1. Introduction

When the skin is injured, it takes a long time to heal, making the affected area more likely to become infected with pathogenic microbes. Therefore, immediate external assistance is required to promote and accelerate the healing process. Bacteria are known to cause the most common types of wound infection, including those caused by *Staphylococcus aureus* and *Escherichia coli* [1,2,3]. The most common treatments used to treat wounds are antibiotics, but the emergence of different antibiotic resistance pathways requires new approaches to innovative treatments [4]. There have been numerous studies in recent years that have proven the therapeutic effectiveness of natural remedies for many diseases, especially skin diseases [5,6,7,8,9,10].

Chitosan is an important biocomponent and a sustainable biopolymer with unique physical, chemical and biological properties [11]. This polymer can also form adhesive membranes [12,13]. These properties make chitosan suitable for use as a wound dressing, as it significantly helps reduce healing time and scarring [14].

Tropical regions are home to the herbaceous succulent plant known as *Aloe vera* (*Aloe barbadensis* Miller). The *Aloe vera* leaves contain anthraquinones, polysaccharides, glycoproteins and a clear mucilaginous gel that is used topically to treat burns and wounds on the skin. *Aloe vera* leaf extract has biological activity and antioxidant [15], anti-inflammatory and healing properties [16]. The presence of glucomannan is primarily linked to the healing effect because it promotes fibroblast growth and activity in the skin, which enhances the ability of cells to produce and secrete collagen [17,18,19].

*Eucalyptus* belongs to the Myrtaceae family. *Eucalyptus* extracts have several pharmacotherapeutic activities, including anti-inflammatory, antioxidant activity, decongestant, and antibacterial properties. The use of essential oils in superficial skin treatments is undermined by allergic skin responses upon direct skin touch. In addition, these volatile non-stable essential oils can oxidize, evaporate, and disintegrate when exposed to heat and light [20]. *Eucalyptus* trees are found in different areas of the world, and their leaves are the main source of essential oils containing terpenoids, phenolics, flavonoids and alkaloids [21]. Recently, nanoparticles were suggested for encapsulation to protect *Eucalyptus* essential oils against evaporation and oxidation, increase the stability of *Eucalyptus* essential oils under harsh environmental conditions, and enhance their water solubility [22].

The rapid development of nanotechnology has revolutionized the design of drug delivery systems, particularly for improving the therapeutic efficacy of bioactive compounds with poor solubility, low stability, or limited bioavailability. Among the various nanoscale carriers investigated, polymeric nanoparticles based on natural polysaccharides such as dextran and chitosan have attracted significant attention due to their inherent biocompatibility, biodegradability, and structural versatility [23]. Polysaccharide-based nanocarriers offer several advantages over synthetic systems, including low toxicity, modifiable functional groups, and the ability to encapsulate a wide range of bioactive molecules, thereby improving targeted delivery and controlled release profiles. These properties make them especially suitable for biomedical applications, including antimicrobial therapy, cancer treatment, and tissue engineering [24].

Dextran and chitosan are complementary polysaccharides that, when combined, create a multifunctional nanosystem with improved physicochemical and biological properties. Dextran is a neutral, hydrophilic polysaccharide known for its excellent biocompatibility, non-immunogenicity, and stability under physiological conditions [25]. Importantly, dextran improves circulation time and steric stabilization, while chitosan provides bioadhesion and intrinsic antimicrobial activity, resulting in a synergistic carrier system [26]. Incorporating eucalyptus extract into a dextran–chitosan nanosystem provides a synergistic platform including carrier delivery, intrinsic polymer bioactivity and plant-derived antimicrobial efficacy. This system is superior to liposomes because liposomes have many drawbacks, like toxicity and physical instability. Conversely, the dextran–chitosan nanosystem is flexible and can co-deliver multiple agents (e.g., drugs + plant extracts). This hybrid nanosystem represents a next-generation platform designed to overcome the limitations of conventional nanocarriers while maximizing therapeutic performance.

This study tries to investigate the potential of a nano-formulation of chitosan, dextran sulfate, *Aloe vera,* and encapsulated *Eucalyptus* extract for wound healing in an animal model. The fabricated nano-formulation was characterized by biophysical techniques, such as transmission electron microscopy, scanning electron microscopy, Fourier-transform infrared spectroscopy, dynamic light scattering, in vitro release profiling and antibacterial activity assays and was subsequently evaluated in vivo for its effectiveness in promoting wound healing.

## 2. Materials and Methods

### 2.1. Materials

Acetic acid and low-molecular-weight chitosan (100–300 K MW) were sourced from Sigma-Aldrich, Saint Louis, MO, USA. Dextran sulfate (35–50 K MW) was procured from AppliChem. *Aloe vera* powder was obtained from High Altitude Organics Natural Essentials.

### 2.2. Fabrication of Formulations

**Figure 1:**
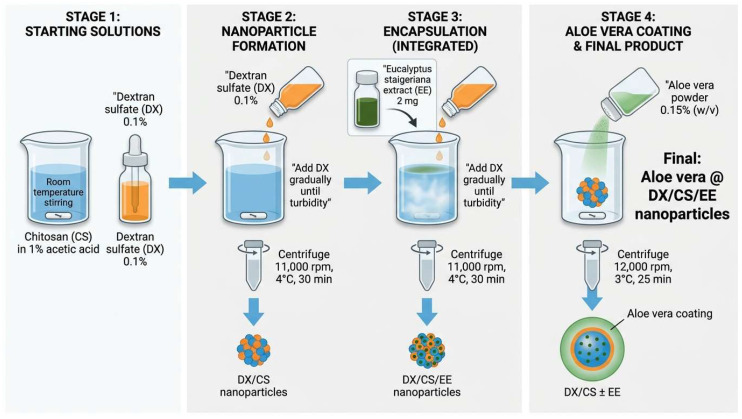
A schematic illustration of the nanoparticle preparation process.

The nanoparticles were prepared at room temperature (Figure 1). A total of 0.05 g of chitosan (CS) was dissolved in 1% acetic acid solution (100 mL) using a magnetic stirrer. Then, a 0.1% dextran sulfate (DX) solution was gradually added until turbidity appeared. Centrifugation was used to extract the nanoparticles (DX/CS nanoparticles) at 11,000 rpm and 4 °C for 30 min. In order to produce a covering of *Aloe vera*, *Aloe vera* powder (0.15% *w*/*v*) was applied to the prepared DX/CS NPs while stirring for 30 min at room temperature. *Aloe vera* @ DX/CS was harvested by centrifugation at 3 °C and 12,000 rpm for 25 min. For *Eucalyptus staigeriana* extract (EE) encapsulation, 2 mg of EE was first dissolved in the CS solution using a magnetic stirrer. Secondly, the dextran sulfate solution was gradually added till turbidity formed, and the DX/CS/EE nanoparticles were collected by centrifugation. For *Aloe vera* coating, *Aloe vera* powder (0.15% *w*/*v*) was added to the prepared DX/CS/EE NPs during stirring (30 min at room temperature), and the sample was then precipitated by centrifugation at 3 °C and 12,000 rpm for 25 min [27].

### 2.3. Gas Chromatography–Mass Spectrometry (GC–MS) Analysis

The chemical composition of the *Eucalyptus* extract was analyzed using gas chromatography (Agilent 8890 GC System, Santa Clara, CA, United States) coupled with mass spectrometry (Agilent 5977B GC/MSD, Santa Clara, CA, United States). Separation was achieved on an HP-5MS fused silica capillary column (30 m length, 0.25 mm internal diameter, and 0.25 μm film thickness). The oven temperature was initially set at 40 °C, then increased to 200 °C at a rate of 4 °C/min, followed by a further rise to 280 °C at 10 °C/min, where it was maintained for 5 min. Helium served as the carrier gas at a constant flow rate of 1.1 mL/min. Samples were injected in splitless mode with an injector temperature of 250 °C. Mass spectra were acquired under electron impact (EI) conditions at 70 eV, scanning a mass range of *m*/*z* 50–500. Compound identification was performed by comparing the obtained spectra with those in the National Institute of Standards and Technology (NIST) mass spectral library.

### 2.4. Characterization of the Nanoparticles

To determine the effectiveness encapsulation of the eucalyptus extract, the extract was extracted from the nano-formula using the centrifuge (VS-18000 M, Vision Scientific Co., Ltd., Daejeon, Republic of Korea, power 220 V/50 Hz) at 12,000 rpm for 30 min at 4 °C. The supernatant containing the extract was collected. A UV-VIS spectrophotometer (JENWAY 6405, Jenway, London, UK) was utilized to determine the entrapment efficiency (EE%) at a wavelength of 465 nm. The EE% was evaluated by the following equation:

EE% = (Total amount of extract − Free amount of extract/Total amount of extract) × 100

The morphological characteristics and dimensional attributes of the synthesized nanoparticles were rigorously investigated using transmission electron microscopy (TEM) (TEM, JEOL JEM.1230, JEOL Ltd., Tokyo, Japan, acceleration voltage: 100 kV) [28]. A 1% phosphotungstic acid solution was used to stain the samples, which were subsequently set on carbon-coated grids, incubated for ten minutes, and ultimately analyzed. A Tescan SEM (Tescan Vega 3 SBU, Brno, Czech Republic) was utilized to scan the surface of the materials using a scanning electron microscope. Lyophilized samples were placed on aluminum microscopy stubs using carbon tape and coated with gold (Au) for 180 s using Quorum Techniques Ltd.’s sputter coater (Q150t, Quorum Technologies, Laughton, UK). The mean size of the samples was measured using dynamic light scattering (DLS). The Nano ZS90 Zetasizer (Malvern Panalytical, Malvern, UK) was used for measurements, which were conducted at room temperature. Using FT-IR NICOLET 6700 (Thermo Scientific spectrometer, Thermo Fisher Scientific, Hemel Hempstead, UK), the FTIR spectra of the lyophilized samples (10 mg) were obtained. Pellets were created by combining each sample with KBr, and the transmittance mode was used to scan the spectra at a resolution of 4 cm^−1^ in the 400–4000 cm^−1^ range. Area normalization was applied to the data.

### 2.5. In Vitro Release Study

The release of EE from the nanoparticles was investigated using the dialysis technique [29]. Both free EE solution (2 mL) and nanoparticulate loaded with EE nanoparticles were placed in semipermeable cellulose acetate membrane bags (Spectra/P with a molecular weight cut-off of 12000, Spectrum, Canada) [30,31]. After that, all loaded cellulose acetate bags were immersed in PBS solution and stirred magnetically at 40 rpm. Over a period of ten hours, aliquots (2 mL) of the solution were taken at predetermined intervals. At a wavelength of 465 nm, spectrophotometric measurement was carried out to ascertain the concentration of released EE in the solution.

To investigate the mechanism and kinetics of EE release from the prepared nanoparticles, the in vitro release data were fitted into various mathematical kinetic models. Zero-order model (describes a system where the drug release rate is independent of its concentration:

$$Q_{t}=Q_{t}+K_{0}\cdot t$$

where Q_t_ is the cumulative amount of EE released at time t, Q_0_ is the initial amount of drug in the solution (usually Q_0_ = 0), and K_0_ is the zero-order release rate constant.

First-order model (describes a release profile that is concentration-dependent):

$$Q_{t}={ln(Q}_{0})+K_{1}\cdot t$$

where Q_t_ is the remaining amount of extract in the nanoparticles at time t, Q_0_ is the initial amount of drug in the nanoparticles, and K_1_ is the first-order release rate constant.

Higuchi model (describes drug release from a solid matrix via a diffusion process based on Fick’s law):

$$Q_{t}=K_{H}\cdot \;t^{0.5}$$

where Q_t_ is the cumulative percentage of EE released at time t, K_H_ is the Higuchi dissolution constant, and t^0.5^ is the square root of time.

Korsmeyer–Peppas model, a semi-empirical model specifically used to analyze drug release from polymeric matrix systems (like nanoparticles):

$$\frac{M_{t}}{M_{\infty }}=K_{\mathrm{KP}}\cdot t^{n}$$

where $\frac{M_{t}}{M_{\infty }}$ is the fractional release of EE at time t (applied only for $\frac{M_{t}}{M_{\infty }}$ ≤ 60), K_KP_ is the release constant incorporating structural and geometric characteristics of the dosage form, and n is the release exponent indicating the underlying extract release mechanism.

### 2.6. Antibacterial Activity

The antibacterial performance of the prepared formulations was systematically evaluated against *Staphylococcus aureus* (*S. aureus,* ATCC:13565) using the colony-forming unit (CFU) counting method. A standardized *S. aureus* bacterial suspension corresponding to 0.5 McFarland turbidity was prepared and propagated in Mueller–Hinton broth to ensure optimal bacterial growth conditions. To assess the antibacterial efficiency of the samples, 96-well microplates were loaded with 200 µL of the freshly prepared bacterial suspension in the presence of the test formulations, alongside appropriate control groups. The plates were incubated at 37 °C for 24 h to allow bacterial interaction with the formulations. Following incubation, 20 µL aliquots from each well were aseptically transferred and spread onto dried nutrient agar plates. These plates were further incubated at 37 °C for an additional 24 h, after which the resulting bacterial colonies were carefully enumerated. The antibacterial efficacy of the tested formulations was quantitatively determined by comparing the viable colony counts of treated samples with those of the control using the following equation to calculate the percentage reduction in bacterial growth [32].

$$Antibacterial\;efficacy\left(\%\right)=\frac{\left(CFU\;count\;in\;the\;control\;\;group-CFU\;count\;in\;the\;exerimental\;group\right)}{CFU\;count\;in\;the\;control\;group}\;\times 100\%$$

### 2.7. In Vivo Antibacterial Activity

#### 2.7.1. Animals

The in vivo topical antibacterial efficacy of the eucalyptus-based formulation was rigorously investigated using a rat excision wound model. Adult male Sprague–Dawley rats weighing 150–200 g were selected for this study to ensure physiological consistency and reproducibility of the results. The animals were housed under strictly controlled environmental conditions, with unrestricted access to standard food and water, and maintained on a 12 h light/12 h dark cycle at a regulated temperature of 21–24 °C and 40–60% relative humidity. Prior to the commencement of the experimental procedures, all animals underwent a one-week acclimatization period to minimize stress-related variables and optimize experimental reliability. All experimental protocols were conducted in full compliance with ethical standards and were formally approved by the National Organization of Drug Control and Research (NODCAR/I/13/2023). The study strictly adhered to the principles outlined in the United States National Institutes of Health Guide for the Care and Use of Laboratory Animals (NIH Publication No. 85-23, revised 2011). Throughout the experimental period, stringent measures were implemented to minimize pain, discomfort, and distress, ensuring humane handling and ethical treatment of the animals while maintaining the scientific rigor of the investigation [33].

#### 2.7.2. Wound Induction

Rats were anesthetized via intraperitoneal injection of chloral hydrate at a dose of 300 mg/kg. The dorsal region was shaved using electric clippers and disinfected with 70% ethanol. Excision wounds, approximately 2.2 cm in diameter, were created on the dorsal surface using toothed forceps and sharp scissors. The wounds were left completely exposed throughout the study [34,35].

#### 2.7.3. Dosing and Drug Administration

The animals were randomly divided into five treatment groups, each containing six rats (n = 6). Group 1 (CONT-Wounded) received normal saline. Group 2 was treated with chitosan–dextran nanoparticles (DX/CS NPs). Group 3 received chitosan–dextran nanoparticles incorporated with *Aloe vera* (*Aloe vera* @ DX/CS). Group 4 was treated with chitosan–dextran nanoparticles containing *Eucalyptus* extract (DX/CS/EE), and Group 5 received chitosan–dextran nanoparticles loaded with both *Aloe vera* and *Eucalyptus* extract (*Aloe vera* @ DX/CS/EE). All formulations were applied topically to the wound site once daily for 10 consecutive days.

#### 2.7.4. Wound Healing Assessment

Wound contraction was monitored every two days for a period of ten days. The wound area of each rat was measured using a transparent sheet and a marker to trace the wound boundaries. The rate of wound contraction was expressed as the percentage reduction relative to the initial wound size [36,37]. The following formula was used to calculate the percentage of wound contraction:

$$\%\;Wound\;contract=\frac{wound\;area\;on\;day\;0\;-\;wound\;area\;on\;day\;n}{wound\;area\;on\;day\;0}\;\times 100$$

#### 2.7.5. Photographic Analysis

Photographic documentation of wound healing in all treatment groups was performed at two-day intervals using a digital camera (Sony, Tokyo, Japan) to monitor macroscopic changes over time [36].

#### 2.7.6. Histological Analysis

At the end of the 10-day experimental period, animals from each treatment group were humanely anesthetized using halothane, after which the wound tissue was carefully excised for detailed histopathological evaluation. The collected skin specimens were immediately fixed in 10% buffered formalin to preserve tissue architecture and cellular integrity. Following fixation, samples were systematically processed through graded dehydration, cleared, and embedded in paraffin wax. Thin tissue sections approximately 5 µm thickness were then prepared using a microtome and subsequently stained with hematoxylin and eosin (H&E). The stained sections were examined under a light microscope to assess epidermal regeneration, inflammatory cell infiltration, collagen deposition, and overall tissue remodeling, providing comprehensive insight into the histological progression of wound healing across the different treatment groups [38].

### 2.8. Statistical Analysis

All experimental data are expressed as the mean ± standard deviation (SD) to reflect variability and ensure statistical transparency. Comparisons among multiple experimental groups were conducted using one-way analysis of variance (ANOVA) to identify overall differences, followed by Tukey’s post hoc multiple comparison test to determine statistically significant pairwise differences. Statistical significance was defined as *p*-value < 0.05. All statistical analyses and graphical presentations were performed using GraphPad Prism software (version 5, GraphPad Software Inc., San Diego, CA, USA).

## 3. Results and Discussion

### 3.1. Extract Characterization

The GC–MS data presented in the Table 1 reveal a complex phytochemical profile of the analyzed *Eucalyptus* extract, characterized by a predominance of oxygenated monoterpenes and their derivatives. Among the identified constituents, isopulegol (31.42%) and citronellol acetate (23.48%) clearly dominate the composition, indicating that these compounds are the principal contributors to the extract’s chemical identity. This finding aligns with previous studies reporting that *Eucalyptus* species—particularly *Eucalyptus citriodora*—often contain high levels of oxygenated monoterpenes such as isopulegol, citronellal, and citronellol derivatives [39], which play key roles in their biological activity. Secondary constituents detected at notable levels include neoisopulegol (8.83%), ethanol (6.81%), citronellal (6.71%), and citronellol (6.11%), suggesting a balanced distribution between major and intermediate compounds. The presence of citronellal and citronellol derivatives further confirms the citronellal–isopulegol chemotype commonly associated with lemon-scented *Eucalyptus* oils [40]. In addition to these dominant compounds, several minor components such as rose oxide (4.34%), isocaryophyllene (2.69%), and *p*-menth-8-en-3-ol (1.18%) were detected, contributing to potential synergistic effects of the extract. Sesquiterpenes such as caryophyllene (1.09%) and its isomers, along with β-longipinene and ledene oxide, were present in smaller proportions; however, they are known to enhance the stability and biological properties of essential oils. The diversity observed in the chromatographic profile reflects the typical variability of *Eucalyptus* essential oils, which is influenced by species, environmental conditions, and extraction methods [41].

Overall, the composition indicates a strong dominance of terpenoid compounds, particularly oxygenated monoterpenes, which are widely recognized for their antimicrobial, antioxidant, and anti-inflammatory activities. The relatively high proportion of acetate derivatives, such as citronellol acetate, also suggest enhanced fragrance stability and potential industrial applications.

### 3.2. Formulations Characterization

**Figure 2:**
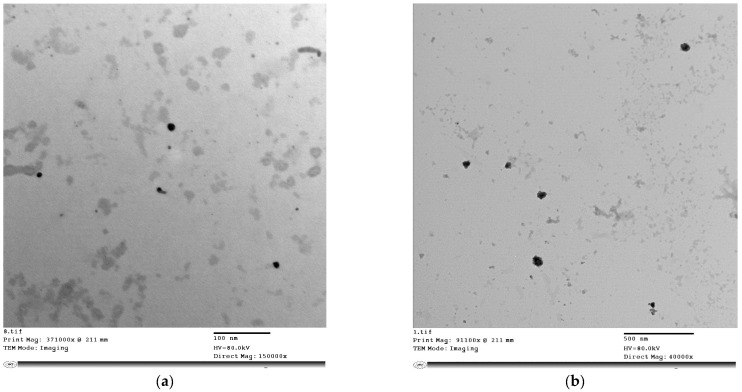

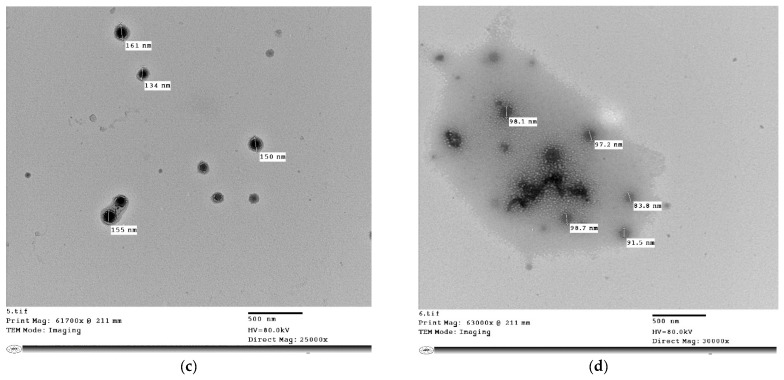
TEM micrographs of (**a**) DX/CS, (**b**) DX/CS/EE, (**c**) *Aloe vera* @ DX/CS and (**d**) *Aloe vera* @ DX/CS/EE NPs.

In this work, eucalyptus oil extract (EE) was loaded into DX/CS nanoparticles and then embedded in an *Aloe vera* gel network, achieving an EE% of 81%. The goal was for *Aloe vera* to build an outer coating layer surrounding the polymeric nanoparticles, resulting in a composite nanostructure. Figure 2a–d display the morphological investigation of the DX/CS, DX/CS/EE, *Aloe vera* @ DX/CS and *Aloe vera* @ DX/CS/EE NPs. The microscopy images of the nanoparticles show unaggregated, homogeneous particles resembling spheres in the nanoscale size range. The *Aloe vera* layer around the polymeric nanoparticles is clearly evident in Figure 2c,d as a light-gray circular outer layer.

**Figure 3:**
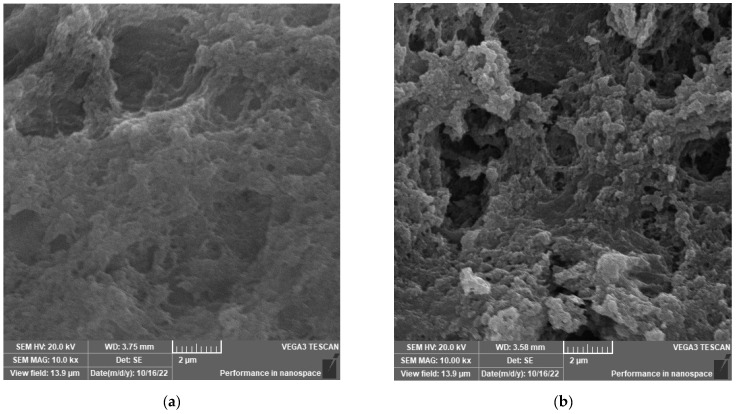

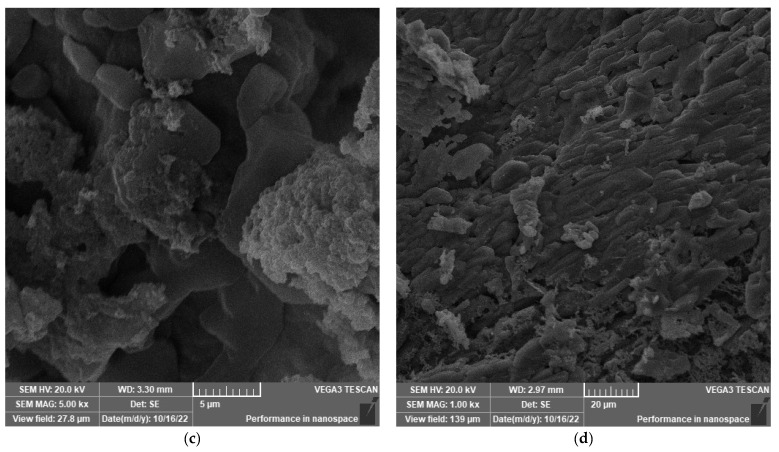
SEM images of (**a**) DX/CS, (**b**) DX/CS/EE, (**c**) *Aloe vera* @ DX/CS and (**d**) *Aloe vera* @ DX/CS/EE NPs.

Additionally, the surface structure of the freeze-dried formulations was examined using SEM. The surfaces of DX/CS NPs and DX/CS/EE were rough and somewhat porous, as shown in Figure 3a,b. The surface smoothness increased, with the appearance of some folds, after the addition of *Aloe vera*, indicating that it might interact physically and ionically with chitosan and dextran sulfate [42] (Figure 3c,d).

**Figure 4:**
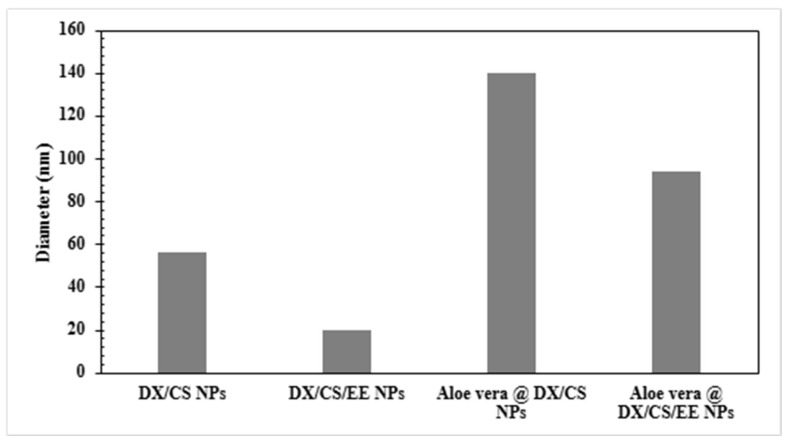
Hydrodynamic diameters of DX/CS, DX/CS/EE, *Aloe vera* @ DX/CS and *Aloe vera* @ DX/CS/EE NPs (PDI ≈ 0.182).

The DLS method was used to measure the zeta potential and particle size of several formulations [43]. The hydrodynamic diameter of both DX/CS NPs and *Aloe vera* @ DX/CS NPs decreased when EE was loaded into them, as seen in Figure 4. This can be explained by the fact that *Eucalyptus* molecules have altered the electrochemistry of the solution, such as the zeta potential, which regulates the internal interactions within the polymer chains [44,45]. The observed decrease in particle size after the incorporation of eucalyptus extract can be attributed to the multifunctional role of phytochemicals (phenolic compounds and small organic molecules) present in the extract. These compounds act as natural surface-active and capping agents, reducing interfacial tension and promoting the formation of smaller nuclei during nanoparticle synthesis. In addition, their adsorption onto the nanoparticle surface inhibits further growth and aggregation, leading to enhanced stabilization and reduced particle size. Furthermore, the interaction between hydrophobic extract components and the chitosan/dextran matrix results in a more compact nanoparticle structure. Similar trends have been reported in essential oil-loaded polymeric nanoparticles, where nanoemulsion formation and surface stabilization contribute to size reduction rather than enlargement [46,47].

**Figure 5:**
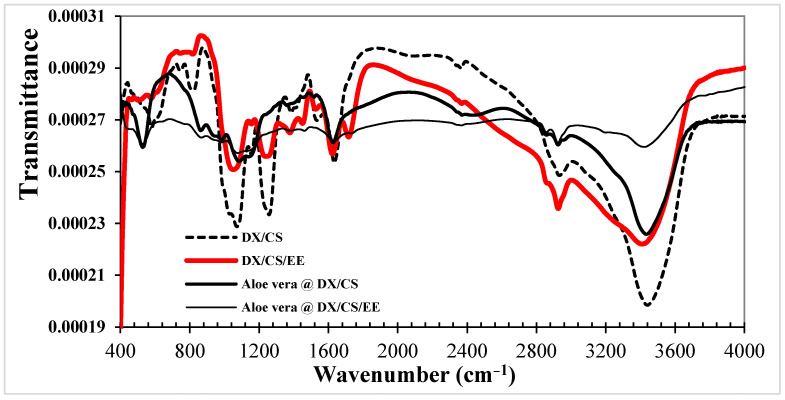
FTIR spectra of DX/CS NPs, DX/CS/EE NPs, *Aloe vera* @ DX/CS NPs, and *Aloe vera* @ DX/CS/EE NPs.

Figure 5 shows the FTIR spectra of DX/CS, DX/CS/EE, *Aloe vera* @ DX/CS and *Aloe vera* @ DX/CS/EE NPs. In the FTIR spectrum of DX/CS NPs, the amine and sulfate absorption peaks were located at 1550 and 1015 cm^−1^. The production of nanoparticles was tracked using the CS peak at 1396 cm^−1^ and the DX peak at 1267 cm^−1^, which are characteristic of these two polymers [48]. This indicates the presence of both polymers and the existence of electrostatic interactions in the final nanoparticle structure. Following the incorporation of eucalyptus into DX/CS nanoparticles, the FTIR spectrum displayed characteristic peaks indicative of eucalyptus extract, indicating the successful incorporation of EE into electroactive polymer matrices. The peak at 1722 cm^−1^ was attributed to carbonyl stretching, while the aromatic ring stretching peak was recorded at 1617 cm^−1^. A peak at 1432 cm^−1^ was associated with carbon–hydrogen bond distortion. The appearance of a strong band at 1060 cm^−1^ was attributed to the stretching of amine groups from N-H [49,50,51]. After applying *Aloe vera* to DX/CS NPs, the peak intensities at 1559 and 1389 cm^−1^ were linked to the symmetrical and asymmetrical stretching of the carboxylate components of *Aloe vera*, respectively [52]. Examination of the FTIR spectra of EE-loaded, *Aloe vera*-coated DX/CS NPs revealed the same findings. The nanoparticulate formulations showed successful interactions between the various ingredients, according to the FTIR data [53].

**Figure 6:**
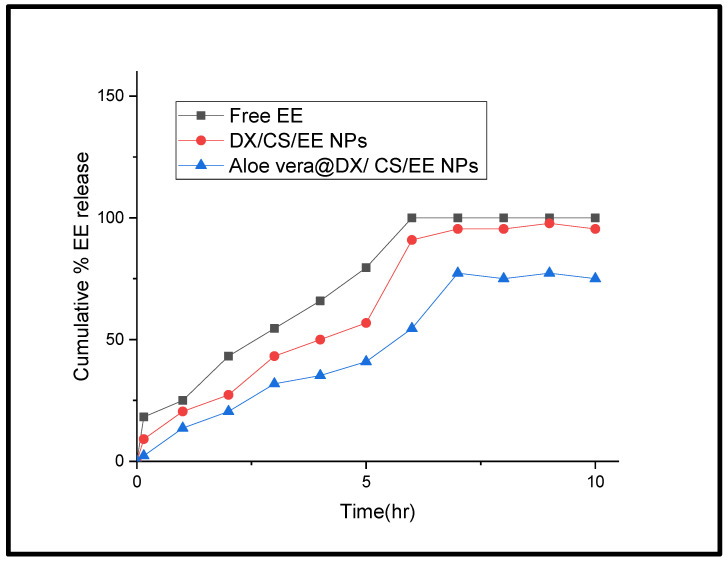
In vitro release profiles of free EE, DX/CS/EE NPs and *Aloe vera* @ DX/CS/EE NPs.

For research purposes, there are ongoing and large-scale efforts aimed at the efficient encapsulation of different drugs at the nanoscale to achieve optimal and well-regulated release profiles for specific drugs [54]. The in vitro release profiles of eucalyptus extract at pH 7.4 were evaluated to assess the performance of the formulated products. The release patterns of EE over a 10 h period before and after incorporation into the produced nanocarriers are shown in Figure 6. The release profile of free EE was the quickest among all tested formulations. The quantity of EE released after three hours from DX/CS NPs was about 43%, while the release from *Aloe vera* @ DX/CS NPs was about 32%, as shown by comparing the patterns of release of DX/CS NPs prior to and following coating with *Aloe vera*. Therefore, after five hours, there was a 15% difference between the amount of EE released from the NPs prior to and following coating with *Aloe vera*. Overall, it is evident that the percentage of EE released from DX/CS/EE NPs and Aloe @ DX/CS/EE NPs differed significantly at 6 h, and the difference increased with time. After seven hours, gradual plateaus were visible in both NP profiles before and after the *Aloe vera* covering. However, only 75% of the EE was released from Aloe @ DX/CS/EE NPs at the conclusion of these plateaus, compared with 95.45% from DX/CS/EE NPs [55,56,57]. To achieve the study goal of improved and prolonged medication efficacy, polymeric nanosystems must have their surfaces modified in addition to their physiochemical properties. This will result in a controlled release pharmaceutical delivery system. Aloe @ DX/CS/EE NPs generated a slower release pattern in comparison to the uncoated one, as clearly shown by the findings. Consequently, applying a layer of *Aloe vera* to the DX/CS NPs produced a more regulated release profile, demonstrating that *Aloe vera* interacted with the other system elements as intended.

As shown in Table 2, the in vitro release data for Free EE and DS/CS/EE NPs exhibited the highest linearity with the Korsmeyer–Peppas model (R^2^ = 0.9892 and 0.9634, respectively). The release exponent (n) values were 0.518 and 0.612, indicating an anomalous (non-Fickian) transport mechanism where drug release is governed by both diffusion and polymer relaxation. On the other hand, Aleo vera @DS/CS/EE NPs followed a zero-order kinetic model (R^2^ = 0.9592), demonstrating a constant and sustained drug release profile over 10 h, which is highly desirable for controlled drug delivery applications.

Furthermore, using the colony-forming unit counting assay, the antibacterial activity of Aloe @ DX/CS NPs and Aloe @ DX/CS/EE NPs against *Staphylococcus aureus* bacteria was evaluated. It was shown that Aloe @ DX/CS/EE hydrogel dramatically reduced the number of bacterial colonies. Aloe @ DX/CS/EE NPs has a much greater bactericidal effect (40%) than Aloe @ DX/CS NPs, as indicated in Table 3. There are several processes that may contribute to the work of the nanoparticles, such as electrostatic contact with the bacterial cell wall, destruction of bacterial DNA, inhibition of protein synthesis, production of reactive oxygen species, or denaturation of proteins and enzymes [58,59]. These results demonstrate that the Aloe @ DX/CS/EE NPs nano-hydrogel has remarkable anti-oxidizing properties, which enhance its antibacterial activity and potentially promote wound healing.

The antibacterial rate of 40% is relatively modest when compared to highly potent antibacterial nanomaterials, such as metal-based nanoparticles. Such high percentages are often reported for systems specifically designed for aggressive bactericidal action rather than for combined therapeutic functionality [60]. Modern hydrogel-based dressings are designed to achieve a balance between biocompatibility, controlled drug release, anti-inflammatory properties, and moderate antimicrobial effects rather than complete bacterial eradication. In this context, even partial antibacterial activity can contribute meaningfully to reducing microbial burden and preventing infection progression. Indeed, nanoparticle- and extract-based systems often exhibit moderate antibacterial effects, but their efficacy improve through sustained release, local accumulation, and synergistic mechanisms at the wound site [61,62]. Furthermore, extract-based formulations, including eucalyptus, are known to provide biological activity that extends beyond direct bacterial killing, such as anti-inflammatory and antioxidant effects, which are equally critical for wound healing. Therefore, while a 40% antibacterial rate may appear limited in isolation, it can still be considered acceptable as part of a multifunctional nanosystem intended for integrated wound management [63].

### 3.3. In Vivo Study

**Figure 7:**
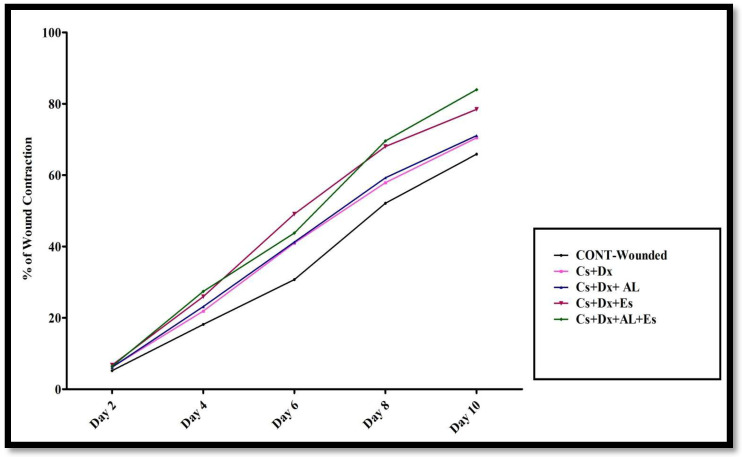
Wound shrinkage percentage in the five treatment groups during the ten-day treatment period with nanoparticles.

**Figure 8:**
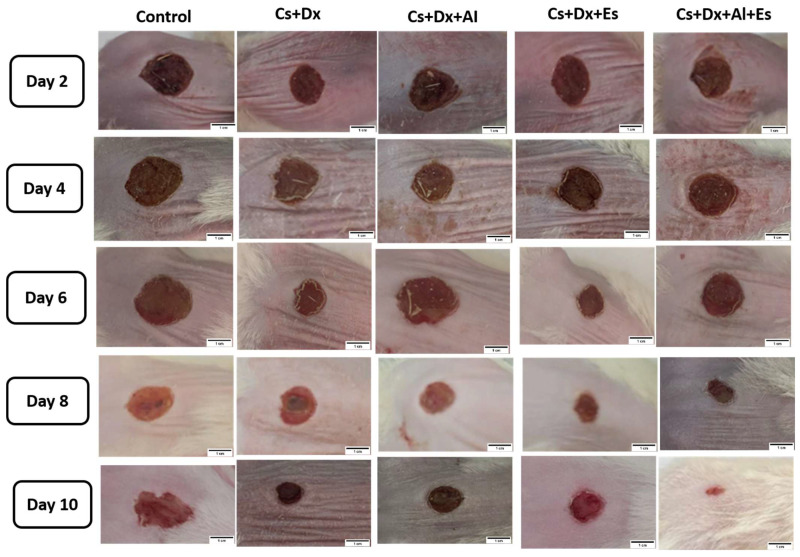
Photographic images showing healing changes at the site of a skin wound in different groups over 10 days of treatment, scale bar =1 cm.

**Figure 9:**
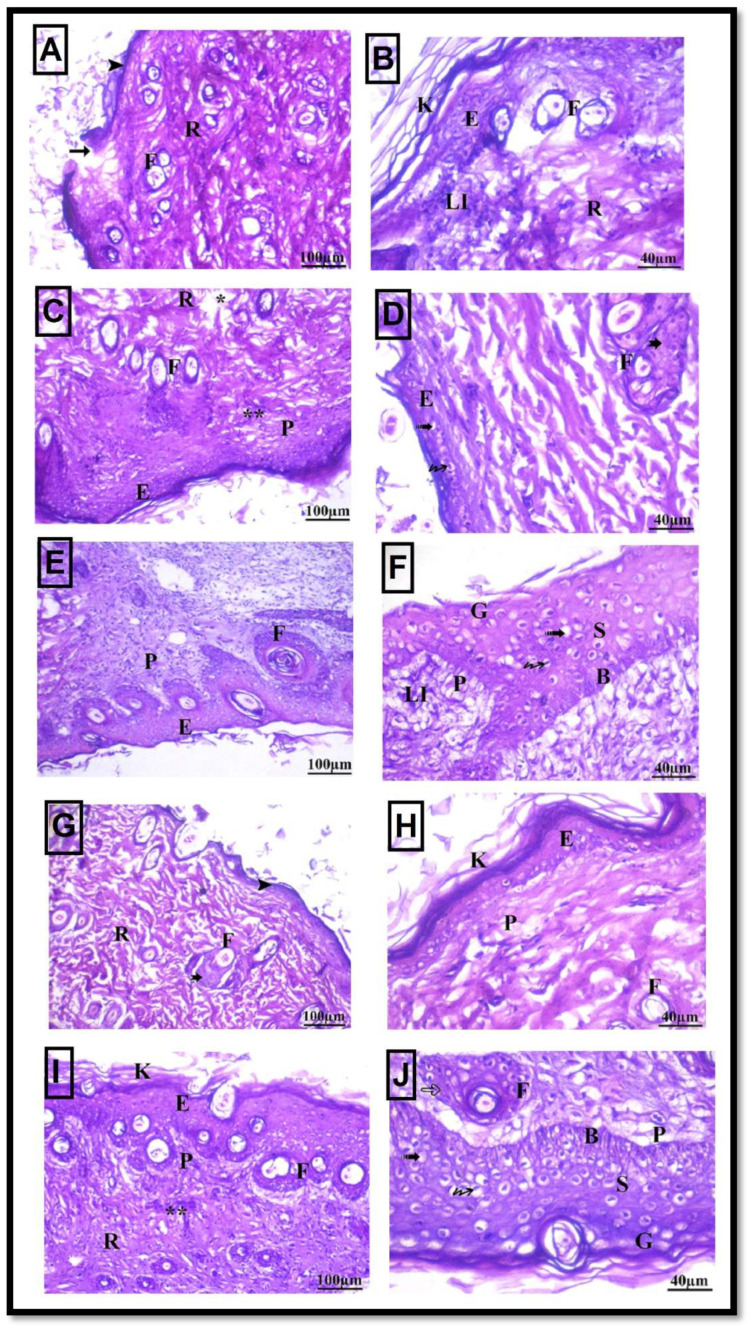
Histological alterations in skin tissue from the CONT-Wounded (**A**,**B**), chitosan + dextran (**C**,**D**), chitosan + dextran + *Aloe vera* (**E**,**F**), chitosan + dextran + *Eucalyptus* (**G**,**H**), and chitosan + dextran + *Aloe vera* + *Eucalyptus* (**I**,**J**) groups (magnification ×40 and ×100). Thin epidermis (arrowhead), vacuolation (dotted arrows), peri-halo cells (wavy arrow), sebaceous glands (notch arrow), epithelial hyperplasia (hollow arrow), empty area (*), lymphocyte invasion in the papillary layer (**), hair follicles (F), hyperkeratosis (K), stratum basal (B), stratum spinosum (S), stratum granulosum (G), papillary layer (P), reticular layer (R), epidermal layer (E), and mononuclear cell infiltration (LI).

Figure 7, Figure 8 and Figure 9 present the findings of the in vivo antibacterial assessment using the rat excision wound infection model. As shown in Figure 7, the percentage of wound contraction was monitored at two-day intervals over the ten-day treatment period. Progressive wound closure was observed across all groups, with variations in the rate and extent of contraction among the different formulations.

From day 2 through day 10 post-injury, the *Aloe vera* @ DX/CS/EE NP group demonstrated a significantly greater wound contraction rate compared with the CONT-wounded group (*p* < 0.05). By day 10, the mean percentage of wound contraction was highest in the *Aloe vera* @ DX/CS/EE NP group (27.45%), followed by the DX/CS/EE NPs (19.18%), *Aloe vera* @ DX/CS NPs (18.12%), and DX/CS NPs (7.03%) groups, relative to the CONT-wounded group.

Additionally, Figure 8 displays photos of the wound healing progress in the various groups during the course of the 10-day treatment.

Figure 9 illustrates the histological analysis of skin tissues collected from the afflicted area in each of the five groups ten days following nanoparticle therapy.

Microscopic examination of skin sections from the CONT-wounded group revealed extensive epithelial disruption. Several areas were covered by a thin epidermis, while others showed complete loss of the epithelial lining. The dermis exhibited irregular vacant spaces between thick collagen bundles within both the papillary and reticular layers. Granulation tissue formation with marked mononuclear cell infiltration was observed. Hair follicles and sebaceous glands were absent beneath the wound area. The epidermis appeared thin, with vacuolated cells and was covered by a thick keratin layer. Additionally, the dermo–epidermal junction was flattened and poorly defined. Displaced sebaceous glands lacking association with hair follicles were occasionally detected (Figure 9A,B).

In rats treated with chitosan + dextran (DX/CS NPs), partial histological improvement was observed. Some regions exhibited a thin, vacuolated epidermis with peri-halo cells. The papillary dermis showed lymphocytic infiltration and dense collagen deposition. Numerous hair follicles lacking associated sebaceous glands were present within the dermis. Keratin lamellae were absent in most areas, although focal regions demonstrated relatively normal epidermal thickness. Collagen fibers were evident within the reticular layer, with reduced interstitial spaces (Figure 9C,D).

Similarly, the chitosan + dextran + *Aloe vera* group demonstrated improved epidermal architecture, with identifiable stratum basale, stratum spinosum, and stratum granulosum layers. Collagen bundles were arranged in multiple directions with minimal separation. Hair follicles were visible within the dermis, accompanied by reduced inflammatory cell infiltration. However, the epidermal surface remained only partially restored, with variable thickness across sections (Figure 9E,F).

In the chitosan + dextran + *Eucalyptus*-treated group, moderate improvement was observed. Certain areas displayed an intact epidermis with vacuolation and a well-formed stratum corneum composed of multiple layers of flattened, non-nucleated keratinized cells. However, some regions still exhibited a thin epidermis. Cutaneous appendages, including hair follicles and sebaceous glands, were frequently detected. The papillary and reticular dermal layers appeared less organized (Figure 9G,H).

In contrast, the chitosan + dextran + *Aloe vera* + *Eucalyptus* group showed marked histological recovery. Most examined areas demonstrated a well-organized and thick epidermis with clearly defined stratum basale, stratum spinosum, and stratum granulosum layers, along with a mature stratum corneum. Dermal organization was largely restored, and inflammatory cell infiltration was minimal. Hair follicles were evident, with some showing epithelial hyperplasia, and sebaceous glands were associated with developing follicles. Overall, this group exhibited the most advanced structural regeneration (Figure 9I,J).

The integration of chitosan, dextran sulfate, *Aloe vera*, and *Eucalyptus* extract into a nanosystem represents a strategically designed approach grounded in their complementary physicochemical and biological attributes. Chitosan is highly effective biomaterial for wound management due to its biocompatibility, biodegradability, intrinsic antimicrobial activity, and capacity to promote cellular adhesion and tissue regeneration, thereby serving as an excellent matrix for hydrogel-based dressings [64,65]. Dextran sulfate further reinforces this platform by enhancing hydrophilicity and structural integrity, facilitating the formation of a stable three-dimensional network capable of sustained drug release and maintaining an optimal moist environment essential for efficient tissue repair. The incorporation of *Aloe vera* introduces additional therapeutic functionality, including anti-inflammatory, antimicrobial, and regenerative effects, which have been shown to significantly accelerate wound closure when combined with chitosan-based systems [66,67]. Concurrently, *Eucalyptus* extract contributes potent antibacterial activity against common wound pathogens, while its nanoencapsulation enhances stability, bioavailability, and therapeutic efficacy [68]. Collectively, the synergistic integration of these components within a nanostructured hydrogel enables controlled bioactive release, effective infection control, and improved tissue regeneration, supporting its potential as an advanced multifunctional wound dressing system [69].

## 4. Conclusions

The present study demonstrates a notable innovation through the development of a fully biocompatible, plant-based nanohybrid system integrating dextran sulfate, chitosan, *Aloe vera*, and *Eucalyptus staigeriana* extract. Unlike conventional nanoparticle systems that often rely on synthetic chemicals or metallic agents, this formulation adopts a green nanotechnology approach, utilizing natural polysaccharides and phytochemicals to achieve multifunctional performance. The incorporation of a polyelectrolyte complex (dextran sulfate/chitosan) combined with *Aloe vera* coating and extract encapsulation, represents a strategic design to simultaneously enhance stability, controlled release, antimicrobial activity, and wound healing potential.

From a quantitative perspective, the nanosystem exhibited a uniform spherical morphology at the nanoscale (~90 nm), as confirmed by electron microscopy, and successfully achieved encapsulation of eucalyptus extract, as validated by FTIR analysis. Functionally, the system demonstrated an antibacterial efficiency of approximately 40% against Staphylococcus aureus, indicating moderate antimicrobial activity. Furthermore, in vivo animal studies revealed a measurable improvement in wound healing efficacy, suggesting that the system effectively supports tissue regeneration beyond its antibacterial function. Despite these promising findings, several limitations should be acknowledged. First, the moderate antibacterial rate (40%) may be insufficient for severe or highly infected wounds, particularly those involving multidrug-resistant bacteria. Second, long-term evaluations of toxicity, degradation behavior, and clinical safety remain unexplored, although these are critical factors for translation into medical applications. Finally, the absence of mechanistic studies explaining antibacterial action.

Future work will focus on optimizing formulation parameters, expanding antimicrobial spectrum, investigating mechanistic pathways, and advancing in vivo validation. Additionally, the development of stimulus-responsive hydrogel systems and scalable production strategies will be essential to facilitate clinical translation of this promising natural polysaccharide-based nanosystem.

## Figures and Tables

**Table 1:** Compounds detected in *Eucalyptus* extract using gas chromatography.

Peak	RT	Area	Area Sum %	Component Name
1	0.706	14,554,132.55	6.81	Ethanol
2	2.398	2,119,596.6	0.99	*O*-Methyl catechol
3	2.746	9,279,526.81	4.34	Rose oxide
4	3.036	1,652,394.84	0.77	*trans*-*p*-2-Menthen-1-ol
5	3.602	67,174,513.14	31.42	Isopulegol
6	3.731	18,874,906.5	8.83	Neoisopulegol
7	3.918	2,513,439.15	1.18	*p*-Menth-8-en-3-ol
8	4.04	1,562,391.08	0.73	2,6-Dihydroxybenzoic acid, 3TMS derivative
9	5.623	13,068,718.04	6.11	Citronellol
10	6.215	2,318,439.99	1.08	Methyl citronellate
11	6.441	1,462,684.6	0.68	Isopulegol acetate
12	7.335	1,541,708.52	0.72	Pulegone
13	8.166	14,341,889.85	6.71	Citronellal
14	8.591	50,194,425.88	23.48	Citronellol acetate
15	9.266	2,328,079.03	1.09	Caryophyllene
16	9.331	2,232,300.88	1.04	*cis*-Jasmone
17	9.53	5,747,806.78	2.69	Isocaryophyllene
18	10.251	1,437,238.1	0.67	*β*-Longipinene
19	11.745	1,377,336.85	0.64	Ledene oxide-(II)

**Table 2:** In vitro drug release behaviors and release kinetics of free EE, DX/CS/EE NPs and *Aloe vera* @ DX/CS/EE NPs.

Samples	Zero-Order (R^2^)	First-Order (R^2^)	Higuchi (R^2^)	Korsmeyer–Peppas (R^2^)	Release Exponent (n)	Best-Fit Model
Free EE	0.8847	0.8252	0.9416	0.9892	0.518	Korsmeyer–Peppas
DX/CS/EE NPs	0.9323	0.8872	0.9419	0.9634	0.612	Korsmeyer–Peppas
Aloe @ DX/CS/EE NPs	0.9592	0.9372	0.9019	0.9482	0.852	Zero-order

**Table 3:** Antibacterial activity of Aloe @ DX/CS NPs and Aloe @ DX/CS/EE NPs against *Staphylococcus aureus* bacteria.

Samples	Number of Bacterial Colonies (cfu) at Dilution Factor 10^−4^	Antibacterial Rate (%)
Blank Control	78 ± 4	-
Aloe @ DX/CS NPs	72 ± 7	6
Aloe @ DX/CS/EE NPs	38 ± 3	40

## Data Availability

The datasets used and/or analyzed during the current study are available from the corresponding author on reasonable request.
